# Effects of a cultural nursing course to enhance the cultural competence of nursing students in Korea

**DOI:** 10.3352/jeehp.2019.16.39

**Published:** 2019-12-27

**Authors:** Hae Sook Park, Hee Jung Jang, Geum Hee Jeong

**Affiliations:** 1 Department of Nursing, Dongyang University, Yeongju, Korea; 2 School of Nursing and Research Institute in Nursing Science, Hallym University, Chuncheon, Korea; The Catholic University, Korea

**Keywords:** Cultural competency, Nursing students, Republic of Korea, Universities

## Abstract

**Purpose:**

With Korea’s recent rapid change into a multicultural society, cultural competence is being emphasized as a core nursing competency. This study investigated the effects of a cultural nursing course that aimed to enhance the cultural competence of nursing students in Korea.

**Methods:**

This was a single-group pre- and post-comparison study. The subjects were 69 nursing students at Dongyang University who attended a cultural nursing course in 2015, of whom 62 students responded to the survey. The 13-week cultural nursing course was held for 2 hours a week. The methods of the course included small group activities, discussions and presentations, experiential learning, reflective activities, and lectures. Nursing students’ cultural competence was measured pre- and post-course with the Cultural Competence Scale for Korean Nurses, which contains 33 items scored on a 7-point Likert scale.

**Results:**

After completing the cultural nursing course, students’ total cultural competence scores increased, as did their scores in each category (cultural awareness, cultural knowledge, cultural sensitivity, and cultural skills) (P<0.001). There was no significant difference in cultural competence by gender (P<0.001).

**Conclusion:**

This cultural nursing course was found to be effective in enhancing the cultural competence of nursing students. Therefore, the educational program developed in this study can be extended to other university-level nursing programs in Korea.

## Introduction

### Background

In Korea, the number of foreign workers, marriage immigrants, international students, and North Korean defectors increased to 2.18 million by 2017 [1]. Furthermore, since 2009, the Korean government has allowed medical institutions to recruit foreign patients, and from 2009 to 2018, the cumulative number of foreign patients reached 2.26 million. The annual number of foreign patients increased from 321,574 in 2017 to 378,967 in 2018 [2]. Therefore, it is important for Korean nurses to be able to understand the diverse cultural background of foreign patients and their families regarding medical care to be able to provide them with comprehensive and holistic nursing care [3]. Cultural nursing competence refers to the ability of nurses and health care providers to understand and respect people with diverse cultural backgrounds and lifestyles, and to provide high-quality patient- or care recipient-centered nursing services [4]. When caring for culturally diverse patients, nurses should incorporate cultural sensitivity into their approach. Giger and Davidhizar [5] in 2008 suggested in their transcultural nursing model that 6 cultural phenomena—communication, space, social organization, time, environmental control, and biological variation—should be considered when subjects are assessed. In some countries that have been multicultural societies for a relatively long time, the importance of nurses’ cultural competence has been fully emphasized. In contrast, cultural competence has not been included as a topic in the curriculum of a number of nursing schools in Korea, despite Korea’s rapid change into a multicultural society [6]. Furthermore, a cultural nursing curriculum has not been included as part of the accreditation of nursing schools by the Korean Accreditation Board of Nursing Education [6,7]. Therefore, nursing schools need to provide opportunities for students to critically discuss their understanding of how to care for foreign patients with cultural diversity. It is necessary to adopt a curriculum that encourages students to explore the role of ethnicity, gender, class, sexuality, and age in nursing care situations for people with a multicultural background [8].

### Purpose

This study aimed to verify the effect of a cultural nursing course on nursing students’ cultural competence. The specific goals of this study were as follows: first, to measure whether students’ cultural nursing competence scores improved after completing cultural nursing coursework; second, to measure differences in cultural nursing competence by gender; and third, to determine students’ overall satisfaction with the course.

## Methods

### Ethics statement

The author (H.S.P.) explained the purpose of the study, the anonymity and confidentiality of the study participants, the use of data for research purposes only, and the ability to choose to withdraw at any time during the survey to the participating students. Informed consent was obtained from the students who wanted to participate.

### Study design

This is an interventional study with a single-group pre- and post-test design.

### Subjects

The subjects were all sophomore nursing students at Dongyang University, Yeongju, Korea who attended and completed the “Multicultural Society and Nursing” course as an elective course in their major in 2015. Of the 69 total students, 62 agreed to participate in this survey. The study subjects were limited to students at a single nursing school because of differences in the content of cultural nursing curricula across a variety of nursing schools. Furthermore, it was not possible to establish a control group at the nursing school where the study was conducted because most sophomore students attended this course.

### Sample size

Assuming that the difference between 2 dependent means (matched pairs) would be tested, an adequate sample size was estimated as 45 using the following input parameters according to Cohen’s power analysis: effect size (D), 0.5; α error probability, 0.05; power (1-β probability), 0.75; and allocation ratio (N2/N1), 2 [9]. Therefore, the sample size was sufficient to estimate the difference of 2 means.

### Measurement tools

The Cultural Competence Scale for Korean Nurses, developed by Chae [10] in 2013, was used as a measurement tool with the original author’s permission. This tool was designed to measure respondents’ cultural nursing competence, understood as the ability to provide patients nursing services that fit the respondents’ cultural values and beliefs. It composed of 4 main elements: cultural awareness, cultural knowledge, cultural sensitivity, and cultural skills. Cultural awareness refers to the recognition of value differences between oneself and individuals from another cultural background and to determine whether one holds any stereotypes and prejudice towards the other culture [11]. Cultural knowledge refers to understanding the social, physical, and biological differences between cultures regarding health beliefs, health practices, disease prevalence, and disease treatment [11]. Cultural sensitivity is the ability to show openness, interest, understanding, empathy, and willingness and ability to communicate with those from different cultures [12]. Cultural skills refer to the ability to communicate, collect data, and make cultural assessments related to the health of the subject [11]. This tool consists of 33 items divided into 4 categories: cultural awareness (6 items), cultural knowledge (7 items), cultural sensitivity (12 items), and cultural skills (8 items) (Supplements 1–3). The average scores of items were used to make comparisons of means. In this instrument, the higher the score, the higher the cultural competence. The internal consistency of the original tool was evaluated using Cronbach’s α, with a value of 0.932. The Cronbach’s α values for the 4 categories were as follows: cultural awareness, 0.905; cultural knowledge, 0.907; cultural sensitivity, 0.921; and cultural skills, 0.879 [10]. In the present study, the overall Cronbach’s α value was 0.900, and the values of the 4 categories were as follows: cultural awareness, 0.763; cultural knowledge, 0.738; cultural sensitivity, 0.854; and cultural skills, 0.817.

### Other survey items

In addition to the measurement tool, demographic information was also obtained. Furthermore, the students were asked an open-ended question on their satisfaction with the course.

### Development and operation of a cultural nursing course at Dongyang University

Two authors (H.S.P. and G.H.J.) have been involved in the development of the multicultural nursing course at Dongyang University for more than 5 years. They first published a textbook entitled “Transcultural nursing” to enhance nursing students’ cultural competence. Based on this textbook, they constructed the content of the course, which focused on cultural awareness, cultural knowledge, cultural sensitivity, and cultural skills as the core elements of cultural competence. The theoretical background of course development was based on the transcultural nursing model of Giger and Davidhizar [5] in 2008, which stresses 6 cultural phenomena in patient assessments and interventions: communication, space, social organization, time, environmental control, and biological variation [5]. The developed course was finalized by verifying its content validity through the consensus of 3 professors of nursing (H.S.P., H.J.J., and G.H.J.) (Table 1). The duration of the cultural nursing course was 2 hours a week for 13 weeks. More specific content on the implementation of the course is presented in Supplement 4.

### Data collection

From March to June 2015, response data were collected from sophomores who attended in the cultural nursing course. The pre-test survey was conducted before the 13-week course and the post-test survey was done after completion of the 13-week course. Written forms of the survey tools were distributed to the students by trained research assistants. Seven students chose not to participate in the survey, meaning that the participants comprised 62 out of the 69 students. In total, 58 responses were analyzed because 3 students did not respond to any items and 1 student was not present to complete the survey. There were no other inclusion or exclusion criteria.

### Statistical analysis

The reliability of the measurement tool was calculated using Cronbach’s α values. The level of cultural nursing competence was analyzed in terms of mean scores with standard deviation. The paired t-test was used to compare pre- and post-test response results. The difference of the pre- and post-test results by gender was tested with 2-way repeated-measures analysis of variance. IBM SPSS ver. 23.0 (IBM Corp., Armonk, NY, USA) and DBSTAT (DBSTAT Co., Chuncheon, Korea) were used for statistical analyses. Students’ degree of satisfaction was also summarized.

## Results

### General characteristics of the subjects

Forty of the students were women (69.0%), and 18 were men (31.0%). Their mean age was 19.7 years (range, 19–23 years). Thirteen students (22.4%) already had received cultural education, 23 (39.7%) had international travel experience, 7 (12.1%) had volunteered or studied abroad, and 4 (6.9%) had a history of living abroad (Table 2).

### Differences in cultural nursing competence before and after participation in the cultural nursing course

The mean scores for cultural nursing competence before and after students participated in the cultural nursing course were significantly different (P<0.001). The subjects’ cultural nursing competence score increased from an average of 4.77±0.64 to 5.83±0.89. Cultural awareness, cultural knowledge, cultural sensitivity, and cultural skills were also found to be significantly different before and after participation in the course (P<0.001) (Table 3, Dataset 1).

### Differences in cultural nursing competence before and after participation in the cultural nursing course by gender

**Fig. 1.**
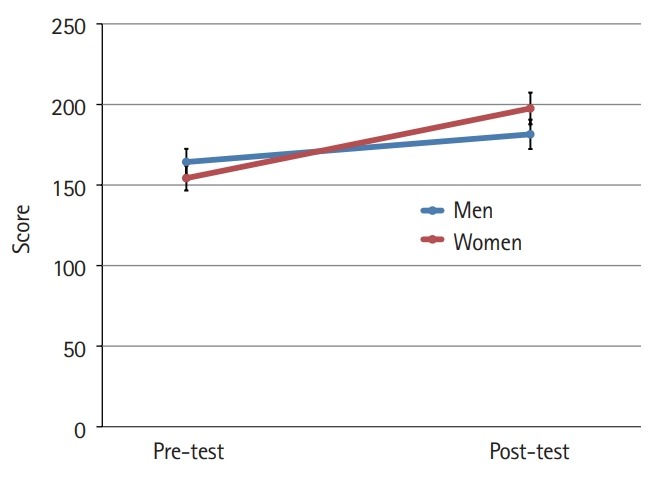
Pre- and post-test comparison of nursing students’ cultural nursing competence according to gender (bar=95% confidence interval).

**Fig. 2.**
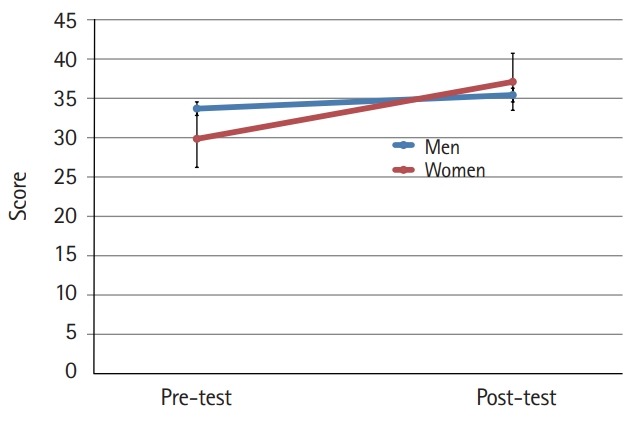
Pre- and post-test comparison of nursing students’ cultural awareness according to gender (bar=95% confidence interval).

**Fig. 3.**
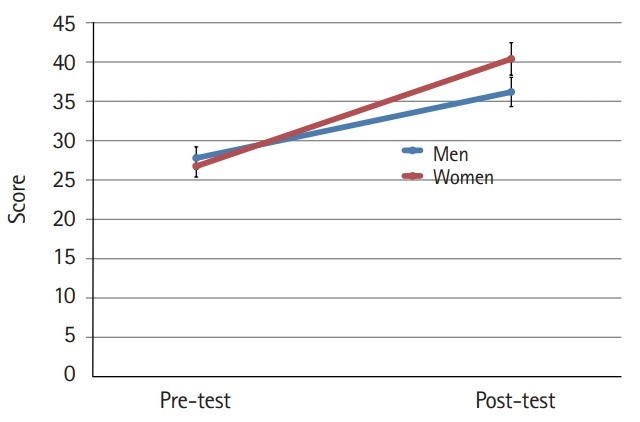
Pre- and post-test comparison of nursing students’ cultural knowledge according to gender (bar=95% confidence interval).

**Fig. 4.**
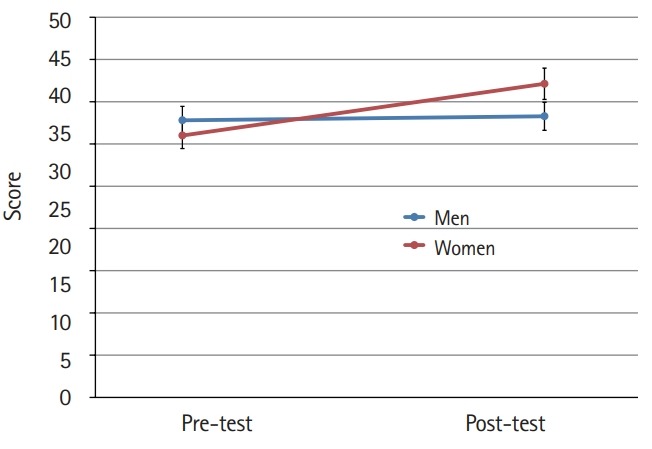
Pre- and post-test comparison of nursing students’ cultural sensitivity according to gender (bar=95% confidence interval).

**Fig. 5.**
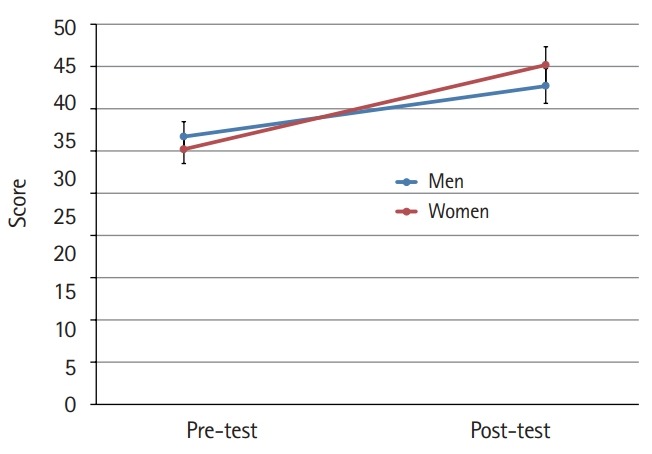
Pre- and post-test comparison of nursing students’ cultural skills according to gender (bar=95% confidence interval).

The mean scores for cultural nursing competence before and after the students participated in the cultural nursing course in both gender groups were different (P<0.001) (Fig. 1). Furthermore, the difference in the mean scores for the 4 category variables was significant (P<0.001). At the pre-test, men’s nursing competence showed a higher score than that of women. At the post-test, the women’s competence scores were higher than those of men, although the difference was not significant at either time point (Figs. 2–5).

### Students’ satisfaction with the cultural nursing course

Students’ satisfaction was measured by a single question: “What do you think about your experiences with this course and the subject in general?” Responses were classified into the following 4 categories: cultural awareness, cultural knowledge, cultural sensitivity, and cultural skills. The main responses were as follows: acquiring knowledge about a variety of countries and cultures (n=22), understanding different cultures through field experiences at a multicultural center (n=15), recognizing the importance of education for cultural nursing (n=12), increasing interest in cultural nursing (n=10), and confidence in communicating with foreigners (n=10) (Table 4).

## Discussion

### Key results

Students’ average scores for cultural awareness, cultural knowledge, cultural sensitivity, and cultural skills all increased after participation in the cultural nursing course. There was no significant difference in these changes according to gender. Therefore, the cultural nursing course developed and applied in this study was found to be effective for improving nursing students’ cultural competence.

### Interpretation

The cultural nursing course developed in this study focused on cultural awareness, cultural knowledge, cultural sensitivity, and cultural skills, which were integrated as a learning strategy for improving cultural competence [11,12]. The course was taught in a way that promoted students’ active participation and critical learning through lectures, small group activities, discussions and presentations, video and movie watching, experiential learning, and a reflection journal. The content and implementation of these activities were found to be effective for enhancing the nursing students’ cultural competence.

The mean scores for the students’ cultural knowledge and skills were lower than those for cultural awareness and sensitivity at both the pre- and post-test time points (Table 3). These results suggest that students are highly sensitive regarding the understanding of diverse cultures and that they respect and accept diverse cultures, but show a comparative lack of accurate knowledge of various cultures and skills in cultural health assessments and communication. After students participated in the course, their cultural knowledge and skills scores significantly improved, to a greater extent than their cultural awareness and sensitivity scores. Furthermore, students’ frequent comments regarding the acquisition of knowledge about a variety of countries and cultures (n=22) support this result. This course was designed not only to enrich nursing students’ knowledge of cultural elements such as communication, social organization, environmental control, and biological differences, but also to improve their communication skills with people from a variety of cultures.

Learning from field visits is important because cultural awareness and cultural sensitivity cannot be strengthened by acquiring knowledge only through classroom training [13]. Therefore, this course was operated in cooperation with a multicultural family support center and comprehensive welfare center in the same geographical location as the university in order to enhance nursing students’ cultural awareness and sensitivity. Foreigners, married migrant women, and North Korean defectors were invited; they gave lectures on their lifestyles and had students try out their traditional attire. Students made foods of other countries, presented their experiences with foreigners through team-based activities, watched related videos, and wrote self-reflection reports. Nursing students have less clinical experience of cultural nursing than nurses; therefore, the effects of cultural education intervention and training may be relatively low [14]. In addition to cultural education, enhanced cultural competence has been found when students have opportunities for cultural experiences, such as meeting and communicating with foreigners from different cultures and visiting or living in foreign countries [7,15]. After participating in the course, students responded to the open-ended inquiry by saying that they were more comfortable with meeting foreigners in person and could understand different cultures more deeply (Table 4). Thus, expanding the opportunities for students to meet and communicate with foreigners, either within the curriculum or through extracurricular activities, will enhance their cultural competence.

### Comparison with previous relevant studies

The above results are in agreement with the meta-analysis of cultural capacity-building curricula and interventions of Gallagher and Polanin [14] in 2015, except for 4 out of 25 studies. The above findings on differences in cultural competence based on category are compatible with the findings of Lee et al. [16] in 2015 the cultural skills and knowledge scores were lower than the cultural awareness and sensitivity scores. In the present study, the effect of the intervention was measured immediately after a 13-week course with 2 hours of class time each week. A study by Lin et al. [8] in 2015 found that nursing students demonstrated an effect on cultural competence for a limited time immediately after receiving cultural competence education, but this effect decreased over time. Therefore, it is difficult to draw any conclusions on the long-term effects of this intervention course on nursing students.

### Suggestion for further study

Because the long-term effects of the interventional course were not investigated, advanced courses for junior and senior students are needed to train the students more intensively. A follow-up check of cultural competence is also required. An educational approach is needed that can sustain and reinforce the concept of cultural competence through its integration throughout the entire curriculum—in both theoretical and practical components—rather than offering a single course to enhance students’ cultural competence. Nurses should provide culturally competent nursing care to people with cultural diversity to improve the quality of care and to enhance health outcomes. Nursing faculty members should continue to develop a variety of educational programs and curricula and to evaluate the effectiveness of these programs in improving students’ cultural competence. The introduction of a cultural competence course into the nursing curriculum would be made more efficient by including it in the evaluation criteria for nursing schools used by the Korean Accreditation Board of Nursing Education. Furthermore, if items measuring cultural competence are included on the Korean nursing licensing examination, many nursing faculty members and students will have a greater interest in this topic.

### Limitations

Because the students were sampled from a single university in Korea using convenience sampling and there was no control group, our results might not be representative of nursing students in general.

### Conclusion

This cultural nursing course was found to be effective in enhancing nursing students’ cultural nursing competence. In particular, students’ knowledge and cultural skills were greatly improved. The importance of nurses’ cultural nursing competence is growing according to the rapid change of Korea into a culturally diverse society. The development and implementation of the cultural nursing course in this study will contribute to improvements in nursing students’ cultural competence.

## Figures and Tables

**Table 1.** An outline of the cultural nursing course at Dongyang University, Korea

Times	Domains	Specific themes and content	Methods
	Pretest	· Self-check of cultural nursing competence	· Questionnaire
1	Introduction	· Orientation	· Lecture
		· Organizing a small group and introducing members	· Group discussion
2	Culture and health	· Understanding culture	· Lecture
		- Definition and attributes of culture	· Group discussion
		- Cultural diversity in nursing	· Group presentation
3		· Understanding disease and health from a cultural perspective	· Lecture
		- Various perspectives of health in different countries	· Group discussion
		- Health beliefs based on culture	· Group presentation
		- The impact of culture on health and disease	
4		· Cultural nursing competence	· Lecture
		- Cultural awareness, knowledge, sensitivity, and skills	· Group discussion
			· Reflective activity
5	Cultural diversity	· Globalization and cultural diversity	· Lecture
		· Domestic multicultural society in Korea	· Group discussion
		- Marriage immigrants, migrant workers, North Korean defectors, foreign students, etc.	· Group presentation
6		· Cultural assessment in nursing	· Lecture
		- Communication, space, social organization, time, environmental control, biological variation	· Group discussion
			· Team report
7-8		· Understanding the different cultures of countries	· Group discussion
		- China, Vietnam, Japan, Thailand, Russia, America, Africa	· Group presentation
			· Team report
9		· Inviting foreigners to experience culture	· Experiential learning
		- Foreigners, married immigrant women, North Korean defectors	· Group discussion
			· Reflective activity
10	Issues in cultural nursing	· Recognizing stereotypes and biases	· Watching movies and videos
		- Multiculturalism in movies	· Group discussion
		· Using medical interpreters	· Reflective activity
		· Ethical decision-making in cultural issues	
11	Cultural nursing intervention	· Communication skills training	· Case study
		- Communicating with people with diverse cultures	· Group discussion
		- Sharing group experiences with married immigrant women, foreign workers, and children of multicultural families	· Group presentation
12		· Health assessment skills training	· Case study
		- Health assessment practice of people with diverse cultures	· Group discussion
			· Reflective activities
13	Wrap-up	· Strategies for improving cultural nursing competence in nursing students	· Group discussion
		· Evaluation of the course, self-reflection	· Group presentation
			· Reflective activities
	Posttest	· Self-check of cultural nursing competence	· Questionnaire
	Term test		

**Table 2.** Subjects’ general characteristics (N=58)

Characteristic	Value
Sex	
Female	40 (69.0)
Male	18 (31.0)
Age (yr)	19.7 (19–23)
<20	24 (41.4)
≥20	34 (58.6)
Experience of receiving cultural education	
No	45 (77.6)
Yes	13 (22.4)
Experience of international travel	
No	35 (60.3)
Yes	23 (39.7)
Experience of volunteering or studying abroad	
No	51 (87.9)
Yes	7 (12.1)
Experience of living abroad	
No	54 (93.1)
Yes	4 (6.9)

Values are presented as number (%) or mean (range).

**Table 3.** Differences in the cultural nursing competence of students before and after participation in the cultural nursing course (N=58)

Variable	Pre-test	Post-test	Difference (pre-post)	Paired t-value	P-value
Cultural nursing competence, total	4.77±0.64	5.83±0.89	-1.06±0.98	-8.27	<0.001
Cultural awareness	5.22±0.84	6.13±1.03	-0.91±1.18	-5.94	<0.001
Cultural knowledge	3.92±0.91	5.61±0.97	-1.69±1.23	-10.47	<0.001
Cultural sensitivity	5.26±0.78	5.99±0.95	-0.73±1.05	-5.28	<0.001
Cultural skills	4.43±0.88	5.57±0.98	-1.44±1.07	-8.091	<0.001

Values are presented as mean±standard deviation.

**Table 4.** Open-ended responses relating to students’ satisfaction after completing the cultural nursing course (N=58)

Category	% (no.)	Specific opinions
Cultural awareness	16.1 (17)	Realizing prejudice towards foreigners and other cultures (8)
		Understanding and respecting cultural differences (5)
		Openness to diverse cultures (4)
Cultural knowledge	30.1 (32)	Acquiring knowledge about a variety of countries and cultures (22)
		Understanding disease, health, and nursing from a cultural point of view (6)
		Lack of knowledge about various cultures (4)
Cultural sensitivity	34.9 (37)	Understanding different cultures through field experiences at a multicultural center (15)
		Recognizing the importance of education for cultural nursing (12)
		Increasing interest in cultural nursing (10)
Cultural skills	18.9 (20)	Confidence in communicating with foreigners (10)
		Improving cultural nursing skills to apply in the actual nursing field (6)
		No opportunity to apply cultural skills to actual nursing care (4)
Total	100.0 (106)	

## Footnotes

**Data availability** Data files are available from https://doi.org/10.7910/DVN/8AO1P5 Dataset 1. Response data of 58 nursing students in Dongyang University to survey questionnaires from March to June 2015.

## Supplementary materials

Data files are available from https://doi.org/10.7910/DVN/8AO1P5

Supplement 1. Survey questionnaire of the pre-test of nursing students’ cultural competence (in Korean).

Supplement 2. Survey questionnaire of the post-test of nursing students’ cultural competence (in Korean).

Supplement 3. English translation of the 33 items on the measurement tool used for the pre-test and post-test.

Supplement 4. Description of the cultural nursing course offered at Dongyang University in 2015.

Supplement 5. Audio recording of the abstract.
