# Supplementary material for: Effects of a cultural nursing course to enhance the cultural competence of nursing students in Korea
Source: J Educ Eval Health Prof. 2019 Dec 27;16:39. doi: 10.3352/jeehp.2019.16.39 (PMC7040427; doi:10.3352/jeehp.2019.16.39)
Supplement: Supplementary file 2 — Supplement 1. Survey questionnaire of the pre-test of nursing students’ cultural competence (in Korean). [file jeehp-16-39-suppl1.pdf]

Supplement 1. Survey questionnaire of the pre-test of nursing students' cultural competence (in Korean).

No.:________________ (1차 설문조사)

설문지 참여 설명문

안녕하십니까?

본 설문은 **다문화사회에서 문화적 다양성을 수용과 적절한 간호제공**을 위해서 대학교육 프로그램에 반영하기 이전에 간호 대학생을 대상으로 **문화적 인식, 지식, 민감성 등 문화역량 정도를 확인하기 위한 조사입니다.**

본 설문은 이번 설문 이후에 **추가로 2차 조사**를 실시할 예정입니다.

귀하가 응답하는 자료는 **비밀이 보장되고 전적으로 연구만을 위해서 사용**되며, 연구조사에 참여 여부로 어떤 이익과 불이익이 있지는 않을 것을 약속드립니다.

귀하의 답변은 본 연구에 귀중한 자료로 활용될 것입니다.

귀한 시간 내어 협조해 주심에 깊이 감사드립니다.

▶ **연구 참여 동의서**

본 연구에 참여를 동의하시는 분은 아래 동의서에 싸인해주시기 바랍니다.

본인은 본 연구목적을 이해하고 자의로 연구 자료수집에 응하기로 동의합니다.

2015년 월 일 서명: ________________

연구자: 동양대학교 간호학부 박혜숙 (연락처: *************)

한림대학교 간호학부 정금희 (연락처: *************) 올림

◆ **다음은 귀하의 문화 관련 인식, 지식, 민감성, 기술과 관련된 내용에 대한 질문으로 귀하에 해당되는 곳에 ‘ V ’표 하시기 바랍니다.**

질문 내용	매우 그렇지 않다			←→			매우 그렇다
1. 나는 문화가 건강과 질병에 대한 인식에 영향을 미친다는 것을 알고 있다.	1	2	3	4	5	6	7
2. 나는 문화에 따라 건강관리행위가 다를 수 있음을 알고 있다.	1	2	3	4	5	6	7
3. 나는 문화에 따라 질병 증상에 대한 표현방법이 다를 수 있음을 알고 있다.	1	2	3	4	5	6	7
4. 나는 문화에 따라 질병의 원인, 치료방법 등에 대한 생각이 다를 수 있다는 것을 알고 있다.	1	2	3	4	5	6	7

5. 나는 외국인 환자의 출신국 의료환경이 간호에 대한 기대 차이를 발생시킨다는 사실을 알고 있다.	1	2	3	4	5	6	7
6. 나는 나의 문화적 배경이 외국인 환자에 대한 내 생각에 어떤 영향을 미치는지 알고 있다.	1	2	3	4	5	6	7
7. 나는 간호사의 성별에 따라 간호행위가 제한되는 문화나 종교신념을 알고 있다.	1	2	3	4	5	6	7
8. 나는 임종간호에 대해 한국인과 다른 요구를 하는 문화나 종교신념을 알고 있다.	1	2	3	4	5	6	7
9. 나는 환자와 간호사가 서로 편안하게 느끼는 거리가 문화에 따라 다르다는 것을 알고 있다.	1	2	3	4	5	6	7
10. 나는 문화에 따라 신체접촉의 의미가 차이가 있다는 것을 알고 있다.	1	2	3	4	5	6	7
11. 나는 문화에 따라 처치나 수술 후 느끼는 통증(민감도)이 다를 수 있다는 것을 알고 있다.	1	2	3	4	5	6	7
12. 나는 문화에 따라 금식에 대한 참을성에 차이가 있다는 것을 알고 있다.	1	2	3	4	5	6	7
13. 나는 치료와 관련된 의사결정 주체가 문화에 따라 어떻게 다른지 알고 있다.	1	2	3	4	5	6	7
14. 나는 외국인 환자의 문화에 맞는 간호를 제공하는 것에 관심이 있다.	1	2	3	4	5	6	7
15. 나는 외국인 환자 간호가 다른 문화를 이해할 수 있는 좋은 기회라고 생각한다.	1	2	3	4	5	6	7
16. 나는 외국인 환자 간호를 할때 상대적으로 시간이 많이 걸리겠지만 기꺼이 간호하고 싶다.	1	2	3	4	5	6	7
17. 나는 문화와 언어의 차이가 있더라도 외국인 환자와 치료적 신뢰관계를 형성할 수 있다고 믿는다.	1	2	3	4	5	6	7
18. 나는 외국인 환자를 간호할 때 내가 대상자와 같은 입장이라면 어떨까라는 생각을 한다.	1	2	3	4	5	6	7
19. 나는 외국인 환자가 우리나라 병원에 왔을 때 경험했던 불편함이나 바라는 점이 무엇인지 알고 싶다.	1	2	3	4	5	6	7
20. 나는 외국인 환자와 대화할 때 시간이 많이 걸려도 참을성을 갖고 들어주려고 할 것이다.	1	2	3	4	5	6	7
21. 나는 외국인 환자를 간호할 때 그 나라 말로 인사말이나 간단한 대화를 시도할 수 있다.	1	2	3	4	5	6	7
22. 나는 다른 문화를 접하는 것에 관심이 있다.	1	2	3	4	5	6	7
23. 나는 외국인 환자에게 효과적인 간호를 제공하기 위하여 필요한 교육이나 훈련을 받기 원한다.	1	2	3	4	5	6	7
24. 나는 학교나 병원 밖 지역사회에서 개최하는 다문화행사에 참여하기 원한다.	1	2	3	4	5	6	7
25. 나는 종교적 신념이나 문화관습 때문에 병원 치료방법을 따르지 않는 외국인 환자의 입장을 이해한다.	1	2	3	4	5	6	7
26. 나는 외국인 환자의 영양을 위해서 문화 특성을 고려한 적절한 식이를 제공할 수 있다.	1	2	3	4	5	6	7

27. 나는 외국인 환자를 간호할 때 그들 문화에서 편안하게 느끼는 거리를 유지할 수 있다.	1	2	3	4	5	6	7
28. 나는 외국인 환자에게 중요한 정보를 설명할 때 가능한 환자의 모국어로 된 문서형태로 정보를 제공할 수 있다.	1	2	3	4	5	6	7
29. 나는 주로 접하는 외국인 환자의 문화를 알 수 있는 자료를 찾아볼 수 있다.	1	2	3	4	5	6	7
30. 나는 외국인 환자와 건강병력을 터놓고 얘기할 수 있을 정도의 치료적 신뢰관계를 형성할 수 있다.	1	2	3	4	5	6	7
31. 나는 외국인 환자와 언어적, 비언어적 의사소통(말투, 몸짓 등)을 적절하게 구사할 수 있다.	1	2	3	4	5	6	7
32. 나는 통역을 이용하여 외국인 환자와 편안하게 의사소통할 수 있다.	1	2	3	4	5	6	7
33. 나는 통역을 이용할 수 없을 때 외국인환자와 필요한 의사소통을 위해 번역기, 통증사정도구 등 필요한 자원을 활용할 수 있다.	1	2	3	4	5	6	7

◆ 다음은 귀하에 대한 질문으로 답해주시기 바랍니다.

1. 성별은? ① 남 ② 여

2. 연령은? 만 세

3. 대학에서 수강과목 중 문화와 관련된 교과목을 수강한 경험이 있습니까?
① 없다 ② 있다 (구체적:)

4. 해외여행 경험이 있습니까?
① 없다 ② 있다 (회)

5. 해외 연수(봉사) 경험이 있습니까? 있다면 어떤 이유인지 체크해 주세요.
① 없다 ② 있다 (구체적)

6. 해외 거주 경험(1개월 이상)이 있습니까?
① 없다 ② 있다 (년 개월)

7. 다문화 관련 교과목을 수강할 경우 학습하기를 기대하는 것은 무엇인지 구체적으로 써주기 바랍니다.
1)
2)

8. 학번: (2차 조사 자료분석 필요함)

▶ 설문지에 성실히 응답하여 주셔서 감사합니다 ◀
