# Supplementary material for: Effects of a cultural nursing course to enhance the cultural competence of nursing students in Korea
Source: J Educ Eval Health Prof. 2019 Dec 27;16:39. doi: 10.3352/jeehp.2019.16.39 (PMC7040427; doi:10.3352/jeehp.2019.16.39)
Supplement: Supplementary file 4 — Supplement 3. English translation of the 33 items on the measurement tool used for the pre-test and post-test. [file jeehp-16-39-suppl3.pdf]

Supplement 3. English translation of the 33 items on the measurement tool used for the pre-test and post-test.

1. I knew culture's impact on perception on health and illness.
2. I knew the differences in health care behavior according to culture.
3. I knew differences in symptoms expression according to culture.
4. I knew the differences in thoughts about causes of illness and treatment according to culture.
5. I knew different expectations about nursing by foreign patient according to their medical environment in their hometown.
6. I knew that my cultural background influence my thought about foreign patient.
7. I knew cultural or religious belief that limits nursing care dependent on nurse's gender.
8. I knew the different expection by foreigners on hospice care according to their cultural or religious belief.
9. I knew that comforable distance between patient and nurse was differenct according to culture.
10. I knew the different meaning of touch according to culture.
11. I knew difference of pain sensitivity according to patient's culture.
12. I knew different enduration to fasting by patient according to culture.
13. I knew that decision maker in the family is different according to their culture.
14. I have an interest in providing culturally appropriate care.
15. I think that caring foreign patient is a good chance to understand different cultures.
16. I am willing to care foreign patients although it takes relatively longer time.
17. I believe that I can build therapeutic relationship with foreign patient although his or her language and culture was different with mine.
18. I sometiems reflect myself if I were foreign patient who receives nursing care from Korean nurse.
19. I would like to know what is the discomfort by foreign patients in his or her visit the hospital or clinic.
20. I have patience to listen to foreign patient even for a long time.
21. I can say simple saluatin to foreign patient with his or her mother language.
22. I have an interest in contacting a variety of cultures.
23. I hope to receive education or training to provide effective care for foreign patient.
24. I would like to participate in the in multicultural activities in the community.
25. I can understand the refusal of treatment by foreign patient due to his or her cultural or religious belief.
26. I can provide the appropriate meal for foreign patient's balanced nutrition considering his or her cultural background.
27. I can keep a proper distance which is comfortable for foreign patient when I care hims or her.
28. I can provide an important document to foreign patient in his or her language.
29. I caan find resources easily about a foreign patient's culture.
30. I can build a therapeutic relationship with foreign patient.
31. I can communicate with foreign patient with not only verbal but also non-verbal communication.
32. I can communicate with foreign patient under help by a translator.
33. I can communicate with foreign patient with translation app or assessment tools in foreign language if a translator is not available.
