# Supplementary material for: Effects of a cultural nursing course to enhance the cultural competence of nursing students in Korea
Source: J Educ Eval Health Prof. 2019 Dec 27;16:39. doi: 10.3352/jeehp.2019.16.39 (PMC7040427; doi:10.3352/jeehp.2019.16.39)
Supplement: Supplementary file 5 — Supplement 4. Description of the cultural nursing course offered at Dongyang University in 2015. [file jeehp-16-39-suppl4.pdf]

Supplement 4. Specific content and operation of cultural nursing course in Dongyang University, Korea

Content of course

The curriculum consisted of the introduction, development, and closing stages. During the introductory stage, students' cultural competence was measured before course start. They were introduced to basic concept and importance of cultural nursing to have interested in the course. In the development stage, learning of content was begun, which consisted of 4 categories such as cultural awareness, cultural knowledge, cultural sensitivity, and cultural skill. Core elements consisted of 6 areas of cultural nursing suggested by Giger and Davidhizar [1] in 2008: communication, space, social organization, time, environmental control, and biological variation. In the closing stage, students shared their own experiences. Their changes of cultural competence was measured. Course work consisted of lectures, discussion, presentation, small group activities, watching movies and videos, experiential learning, and reflective writing activities.

Operation of course

This course is an elective course with 2 credits majors during 13 weeks of each 2 hours a week except 2 weeks of midterm and term exams. In the class, the professor made a lecture on the core concepts. After that, students discussed together in a team consisted of 5–6 students. After watching video or movie on the culturally diverse situations or examples, they discussed on the stereotype or prejudice in a variety of situations in a team. Four foreigners were invited to present their experiences in Korea, who were 1 foreigner who lived in Korea, 2 international marriage migrant women, and 1 North Korean defector. They made lectures on culture, explained the traditional costume and made meal with students. After that, students discussed their experiences in a team. Their experience was shared with other teams. The professor encouraged students to participate in an active discussions. The professor supported active interaction with students who were not active in a team. Students' reflective writing activities about learning in the course were opened and they discussed their opinion how culture affects health assessment or nursing in the last week.

References

1. Giger JN, Davidhizar RE. Transcultural nursing: assessment and intervention. 5th ed. St. Louis (MO): Mosby Elsevier; 2008.
